# Implementation of artificial intelligence-based decision support systems for antibiotic prescribing in hospitals: a Delphi study

**DOI:** 10.3389/fdgth.2025.1555042

**Published:** 2025-04-25

**Authors:** Pinar Tokgöz, Joanna Albrecht, Christoph Dockweiler

**Affiliations:** Faculty of Arts and Humanities, Professorship of Digital Public Health, University of Siegen, Siegen, Germany

**Keywords:** antibiotic prescribing, artificial intelligence, decision support, clinical practice, implementation

## Abstract

**Introduction:**

Numerous initiatives against antimicrobial resistance have been initiated in recent years. Decision support systems (DSSs) based on artificial intelligence (AI) provide new opportunities for automating antibiotic therapy in hospitals. While AI-based DSSs may improve antimicrobial use and patient outcomes and reduce healthcare costs, the challenges associated with their implementation, optimization, and adoption cannot be ignored.

**Methods:**

A Delphi study was conducted to investigate factors influencing the implementation of AI-based DSSs in the hospital setting.

**Results:**

The study included 36 experts with perspectives on the hospital setting and DSS development. A consensus was reached on the importance of 34 factors and the ranking as well as assessment of current realization of implementation factors revealed important starting points for implementation strategies.

**Discussion:**

The study results indicate that whilst there are multiple factors of importance in DSS implementation, some factors, as e.g., promoting application- and user-orientated development of DSSs, establishing user-friendly organizational structures, and fulfilling demands of trust, transparency, and responsibility through sensitization and education on organizational but also legal level should gain more attention. In addition, two factors did not reach a consensus in terms of importance, indicating that it may not be practical to consider all factors of importance when implementing AI-based DSSs in the hospital setting.

## 1 Introduction

Combatting antimicrobial resistance is widely regarded as a priority area in public health and several strategies haven been developed in response (1). One of the main causes of this problem is the over- or misuse of antibiotics (2). For this reason, antimicrobial stewardship programs (ASPs) have been established to optimize antimicrobial usage, including recommendations about strategies for prescribing antibiotics in clinical practice, such as the selection of adequate antibiotics, and the dosage or duration of the therapy (3, 4). The ASP-teams, which are composed of specialists from various clinical fields, like clinicians, pharmacists, hygienists, and managers, are considered fundamental to achieve optimization in the rational use of antibiotics within hospitals (5). A growing amount of evidence shows that ASPs can both optimize the management of infections and reduce the emergence of antimicrobial resistance (6). Nevertheless, a well-known disadvantage of ASPs is the amount of time required to review and document antibiotic therapy (7). There is therefore an urgent need to apply more effective tools to better support clinicians in the complex task of choosing the most appropriate antibiotic treatment. Machine learning techniques and the increasing availability of high-quality, large-scale data offer new opportunities for optimizing antibiotic therapy (8). Thus, decision support systems (DSSs) based on artificial intelligence (AI) use largely automated general learning procedures to identify statistical regularities from the (training) data presented to them and in turn generate predictive probability statements for the occurrence of phenomena (9). The use of AI-based DSSs can be a key factor in improving the results of ASP-teams, considering the multi-user perspective of the problem, the need for knowledge integration from different sources, and the requirement to provide support both for a particular patient and for the whole institution in a coordinated manner. Despite the promising evidence, studies show that there remains some level of inconsistency about the relative merits of AI-based DSSs in influencing practice patterns in hospitals, how to implement them, and what refinements are needed to tailor the systems to local contexts (10). However, the predominant focus on technical prowess during AI development often sidelines considerations for seamless integration into real-world workflows and the practical value of these innovations. If AI-based DSSs are ever to be integrated successfully, it will be essential to establish suitable conditions and develop an adequate strategy for the implementation.

This Delphi study aimed to determine factors that could influence the implementation of AI-based DSSs for antibiotic prescribing in hospitals from the perspective of experts in the field of antibiotic therapy and AI-based DSSs in practice and research.

## 2 Methods

**Figure 1:**
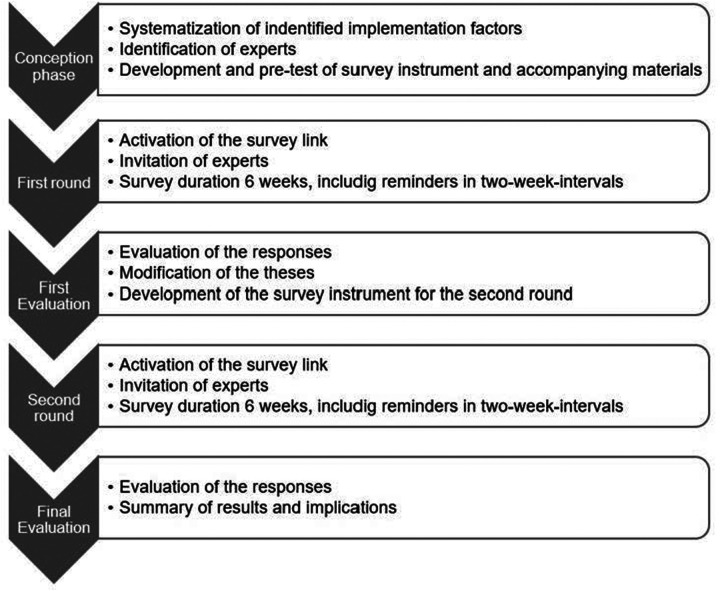
The Delphi process.

A two-round Delphi study was undertaken to establish expert consensus on the importance of factors on DSS implementation for antibiotic prescribing in the hospital setting. The Delphi technique is a research method where sequential surveys are used to gain individual expert opinion across several rounds whereby the anonymized results are provided as feedback to the participants (11, 12). In this sense, from the second round of the survey onwards, the experts make their judgements under the influence of the opinions of the other participating experts. In this respect, the Delphi method represents an iterative procedure in which expert assessments on a specific question are determined with the aim of recording and justifying consensus and/or dissent in the judgements (13). The benefits of this method include the possibility of gaining the perspective of an expert group and build consensus in an area where evidence may be lacking (14). The process of the Delphi is illustrated in the following (Figure 1). Ethics approval to conduct the study was obtained from the University of Siegen, project number: LS_ER_25_2024.

### 2.1 The Delphi instrument

A modified approach was used for this study. The traditional qualitative approach for the first round was discarded (15). Instead, factors for examination were conducted based on two previous studies. The first study involved a systematic review of all literature where implementation factors of AI-based DSSs for antibiotic prescribing in hospitals were described (16). In the second study, a qualitative study of clinicians' perspectives on implementation factors was conducted (17). The preliminary work formed the Delphi instrument used in round one and to provide arguments relating to the factors that could facilitate or hinder the implementation of AI-based DSSs for antibiotic prescribing in hospitals and to which the level of agreement by the participants was acquired. The arguments were grouped into the three domains of the Human-Organization-Technology-(HOT)-fit-model (18), namely human, organization, and technology. Responses were collected with a five-level Likert scale for the evaluation of each thesis and argument: “Disagree” (category response value=1), “rather disagree” (= 2), “rather agree” (= 3), “agree” (= 4) and “I cannot assess” (= 5). An open text box for additional comments related to the topic was provided requiring further investigation in the following round. In the second round, experts were asked to assess the aggregated results of the first round and additionally rank the arguments about the importance for implementation as well as assess the level of realization in current clinical practice and to provide possible interventions for the improvement of current realization. The Delphi instrument was pre-tested for comprehensibility and time required for responses by two people of the target group who did not participate in the main survey. The web-based survey, which was conducted through LimeSurvey was initiated in July and closed in October 2024.

### 2.2 Survey participants and recruitment

The panel was arranged using purposive sampling (19). According to this, the recruitment focused on expertise and diversity of perspectives rather than representativeness and large sample size (20). So, a heterogeneous panel of experts from research and practice has been compiled. For the study, experts were defined as people who are in a position to assess developments in the healthcare sector concerning the use of AI-based DSS for antibiotic therapy in hospitals. The expert status of the practice-related experts was defined by a professional position or clinical expertise in the field of antibiotic therapy (e.g., by working as an ASP-member or corresponding professional responsibility in the field of antibiotic therapy). The expert status of scientific experts is defined by scientific achievements, e.g., through publications, special lectures, third-party funded projects, or a proven field of research related to AI-based DSS in healthcare and/or antibiotic therapy e.g., through position title/affiliation that reflects involvement). Specific knowledge of AI-based DSSs was not required, since the term “AI-based DSS” has been explained in the invitation to the survey. 511 experts were invited from across Germany to take part in the study and invitation emails were sent to the individually identified potential participants. Additionally, reminder emails were sent in two-week intervals to all contacts to incentivize those who have not yet participated to attend the survey.

### 2.3 Consensus and statistical analysis

An analysis of responses was performed after each survey round. As there is no universally defined level of agreement for consensus (19) and a systematic review including 100 Delphi studies found that percent agreement was most frequently used (21), a consensus has been determined according to the rating scale proposed by Meskell et al. (22). The proposed scale is based on preliminary work by de Loë (23), which is also recommended in several studies (24, 25). Consensus is achieved by initially calculating the percentage agreement on items by adding up the case number that achieved the same rating and calculating the percentage. Thus, the level of consensus can be “high”, “moderate” or “low” and the direction of consensus can be “in favor” [+] or “against” [−]. A consensus on an argument or thesis is deemed to have been achieved when the group responses reach the predefined percentage threshold. For example, if more than 70% of participants rate an item in one category (e.g., “strongly agree”) then the item has a high consensus level. Similarly, if less than 50% of participants rate the item in the category “strongly agree” then the item has a low consensus level. If no consensus was reached, the individual agreement values have been reported. In the next round, the factors for which the responses in the first round resulted in dissent or a moderate or weak consensus (±2% distribution around cut-off value) are submitted for renewed assessment. In addition, the first-round analysis included a thematic analysis of the open-text-comments to identify additional aspects for inclusion in the second round or reformulating the arguments. The responses of the participants were analyzed, who fully completed the survey. The results were evaluated using SPSS version 24.0 and descriptive statistics. Group differences in the first round were analyzed between the fields of profession (clinical practice and research) using Mann–Whitney-*U*-tests. The Henry Garret ranking method technique (26) was applied to discover the most important factors influencing the implementation of AI-based DSSs for antibiotic prescription in hospitals.

## 3 Results

77 of the 511 potential participants (15%) invited to take part in the survey accepted the invitation and participated in the first round. 36 of the 77 experts (47%) who initially completed the first round completed the second round. Participant demographics and professional background are presented in Table 1.

**Table 1:** Participants characteristics.

Demographic		Round 1 (*n* = 77)	Round 2 (*n* = 36)
Gender	Female	36.4% (*n* = 28)	33.3% (*n* = 12)
	Male	63.6% (*n* = 49)	66.7% (*n* = 24)
Age	20–29 years	1.3% (*n* = 1)	2.8% (*n* = 1)
	30–39 years	19.5% (*n* = 15)	25.0% (*n* = 9)
	40–49 years	35.1% (*n* = 27)	25.0% (*n* = 9)
	50–59 years	32.5% (*n* = 25)	38.9% (*n* = 14)
	60 years or older	11.7% (*n* = 9)	8.3% (*n* = 3)
Current professional role	Hospital/Health care services (e.g., an ASP-member)	77.9% (*n* = 60)	75.0% (*n* = 27)
	Technology-related hospital services (e.g., staff in the hospital IT)	2.6% (*n* = 2)	–
	Academic/researcher (e.g., in the fields of AI-based decision support systems)	16.9% (*n* = 13)	22.2% (*n* = 8)
	Other (e.g., hospital hygiene)	2.6% (*n* = 2)	2.8% (*n* = 1)
Years of experience	Up to 5 years	13.0% (*n* = 10)	11.1% (*n* = 4)
	Up to 10 years	26.0% (*n* = 20)	27.8% (*n* = 10)
	More than 10 years	61.0% (*n* = 47)	61.1% (*n* = 22)
Level of expertise in the field of antibiotic therapy	Very high	36.4% (*n* = 28)	44.5% (*n* = 16)
	High	42.9% (*n* = 33)	33.3% (*n* = 12)
	Moderate	9.1% (*n* = 7)	5.6% (*n* = 2)
	Low	5.2% (*n* = 4)	8.3% (*n* = 3)
	Non-specialist	6.5% (*n* = 5)	8.3% (*n* = 3)
Level of expertise in the field of AI-based DSSs	Very high	6.5% (*n* = 5)	2.8% (*n* = 1)
	High	10.4% (*n* = 8)	13.9% (*n* = 5)
	Moderate	36.4% (*n* = 28)	44.5% (*n* = 16)
	Low	35.1% (*n* = 27)	30.5% (*n* = 11)
	Non-specialist	11.7% (*n* = 9)	8.3% (*n* = 3)

### 3.1 Potential of AI-based DSSs for antibiotic therapy in hospitals

**Figure 2:**
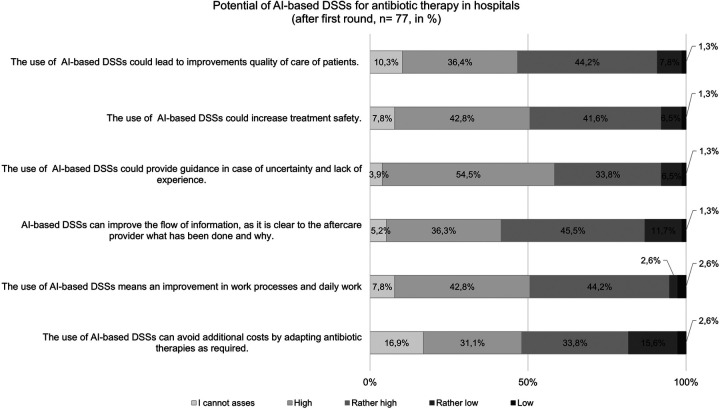
Assessment of the potentials of AI-based DSSs (results from the first round; *n* = 77).

The experts were asked how they would assess the various potentials of AI-based DSSs for antibiotic prescribing in hospitals (Figure 2).

In the first round, the experts agree about the fundamental and divers potentials of AI-based DSSs for antibiotic prescription in hospitals. Thus, the participants grade the potential of AI-based DSSs for providing guidance in case of uncertainty (88%) and improving work processes (87%) as high or rather high. Furthermore, 82% of those surveyed assess the potential of AI-based DSSs for improving the flow of information between providers and thus increasing treatment safety (84%) as high or rather high. The assessment of the potential related to reducing costs is less consistent. Here, 65% of the respondents assume the potential as being high or rather high, whereas 18% assess this potential as being low or rather low and 17% couldńt give an assessment. In the second round, all participants (*n* = 36) agree with the overall picture of the first-round results.

### 3.2 Factors of implementation

The following tables show the key results for each of the arguments that could influence AI-based DSS implementation. The arguments are grouped in the domains of the HOT-fit model, and arranged in ascending order by level and direction of consensus.

Those who took part in the Delphi study are uniformly in favor of the aspect that for implementation of AI-based DSSs data of sufficient quality and their regularly maintenance should be given (Table 2).

**Table 2:** Results for the assessed technology-related theses.

Topics and arguments	Consensus*	Direction
**Technological factors**		
**Clear presentation of the results:** AI-based DSSs should be attractively designed, and recommendations should be clearly prepared and presented.	(+++)	In favor
**Reliable database:** The basis for the beneficial use of AI-based DSSs is data of sufficient quality and its regular maintenance.	(+++)	In favor
**System integration compatibility:** AI-based DSSs should be based on solid data models, interoperable formats, and internationally recognized coding standards so that they can be integrated into any computer system.	(+++)	In favor
**Warning functions:** To provide helpful support in everyday working life, warning functions of AI-based DSSs should be specific.	(+++)	In favor
**Existence of alternative suggestions:** An equivalent treatment alternative should be listed to be able to act, e.g., in the case of intolerances and allergies.	(+++)	In favor
**Data security:** Data protection and security should be regulated in a detailed and generally understandable manner.	(+++)	In favor
**Precise recommendations:** With AI-based DSSs precise recommendations should be made and direct instructions given on what exactly to do.	(+++)	In favor
**Manageable user interface with easy navigation:** AI-based DSSs must be uncomplicated to use and easy to operate.	(+++)	In favor
**Rapid system updating:** AI-based DSSs should be able to be updated quickly.	(+++)	In favor
**Automated data transfer:** AI-based DSSs are most effective and beneficial when their data is transferred automatically from existing hospital information systems.	(+++)	In favor
**Easy access to the system data:** AI-based DSSs should be easily accessible and approachable from all common end devices such as smartphones/tablets.	(+++)	In favor
**Traceability of recommendations:** Users should be provided with comprehensible and expert-curated decision support for possible therapeutic options.	(+++)	In favor
**Completeness of recommendations:** The recommendations should be comprehensive and complete (e.g., dosage recommendation, duration of therapy).	(+++)	In favor
**Easy manual data entry:** It is important that AI-based DSSs are designed in such a way that no programmer is required to insert or modify data.	(++)	In favor
**Individual-specific recommendations:** AI-based DSSs should offer recommendations for specific or individual patient problems.	(+)	In favor

Threshold for consensus in accordance with Meskell et al. (21):

Consensus*:

“High” (+++) >70% in category 4 or 3; >80% in categories 4 and 3.

“Moderate” (++) >60% in category 4 or 3; >70% in categories 4 and 3.

“Low” (+) >50% in category 4 or 3; >60% in categories 4 and 3.

“High” (—) >70% in category 2 or 1; >80% in categories 2 and 1.

“Moderate” (–) >60% in category 2 or 1; >70% in categories 2 and 1.

“Low” (-) >50% in category 2 or 1; >60% in categories 2 and 1.

“Dissent” (x): No unanimous group response.

N_1_ = 77; N_2_ = 36.

^a^ Results of the second round.

In addition, they expect AI-based DSSs to be uncomplicated in use and easy to operate as well as recommendations to be presented clearly and comprehensible. Although expert consensus demonstrates the importance of patient-specific recommendations, several participants highlight the possible conflicts of DSSs to make specific recommendations and at the same time being too difficult to operate with:

“The system would have to be far too complicated and confusing for truly individualized recommendations.” (Participant 19)

The experts are confident that political and legal frameworks are required for establishing AI-based DSSs. They also believe that supporting users in technical matters and maintaining the system is essential for long-term implementation (Table 3).

**Table 3:** Results for the assessed organization-related theses.

Topics and arguments	Consensus*	Direction
**Organizational factors**		
**Hospital's willingness to change:** The willingness of hospitals (or of the decision-makers in hospitals) to integrate new technologies into healthcare is essential for AI-based DSS implementation.	(+++)	In favor
**Restructuring of “traditional” working routines:** AI-based DSSs should be integrated in such a way to support established workflows. A collateral systematic organizational development is therefore required.	(+++)	In favor
**Openness of (medical) teams/units:** The openness of (medical) teams within an organization towards AI-based DSSs can promote its implementation.	(+++)	In favor
**Technical equipment:** Sufficient equipment, e.g., in the form of computers/laptops and PC workstations, is a favorable aspect for the implementation of AI-based DSSs.	(+++)	In favor
**Availability of technical support:** Supporting users in technical matters and the maintenance of the system is essential for implementation.	(+++)	In favor
**Clarification of the legal framework:** Regulatory control (regulation) and clarification of liability issues are essential for long-term implementation.	(+++)	In favor
**Restructuring medical education:** The holistic development of AI skills requires further and advanced training programs as well as focal points in medical education.	(+++)	In favor
**Support from the management level:** Support from clinical leadership/hospital board representatives can facilitate the implementation of AI-based DSSs.	(+++)	In favor
**User participation in the development and implementation phase:** Future users must be involved in the design and implementation of AI-based DSSs.	(+++)	In favor
**Training of potential users:** To increase skills in dealing with AI-based DSSs, sufficient training opportunities should be offered for users as well as ongoing familiarization.	(+++)	In favor
**Overcoming hierarchical structures:** Hierarchical structures and dependence on established standards are an obstacle to the implementation of AI-based DSSs.	(+)	In favor
**Financial incentives:** Increased monetary incentives for the implementation and use of AI-based DSSs in hospitals are essential for successful implementation.^a^	(x)	

Threshold for consensus in accordance with Meskell et al. (21):

Consensus*:

“High” (+++) >70% in category 4 or 3; >80% in categories 4 and 3.

“Moderate” (++) >60% in category 4 or 3; >70% in categories 4 and 3.

“Low” (+) >50% in category 4 or 3; >60% in categories 4 and 3.

“High” (—) >70% in category 2 or 1; >80% in categories 2 and 1.

“Moderate” (–) >60% in category 2 or 1; >70% in categories 2 and 1.

“Low” (-) >50% in category 2 or 1; >60% in categories 2 and 1.

“Dissent” (x): No unanimous group response.

N_1_ = 77; N_2_ = 36.

^a^ Results of the second round.

Moreover, to increase skills in handling AI-based DSSs, sufficient training opportunities and ongoing familiarization should be offered for (potential) users. Beneath that, AI-based DSSs should be integrated in such a way to support established workflows. Therefore, collateral systematic organizational development and overcoming hierarchical structures are required. The respondents agree likewise that the willingness of hospitals to integrate new technologies into healthcare and the support from clinical leaders can facilitate the implementation. There is no consensus on whether financial incentives for hospitals on the decision to implement and use AI-based DSSs could serve as facilitators. In this context, 30.6% of the participants rather disagree and 8.3% disagree, that monetary incentives are essential for the successful implementation of AI-based DSSs in hospitals, whereas 19.4% agree and 38.9% of the participants rather agree.

The experts believe that AI-based DSSs can be implemented successfully in the long term if users perceive an added value of the use and benefit noticeably from it. Promoting skills in operating with AI-based DSSs, openness towards its use, and trust in it are found to favor implementation (Table 4).

**Table 4:** Results for the assessed user-related theses.

Topics and arguments	Consensus*	Direction
**User-related factors**		
**Promoting of competencies in operating with AI-based DSSs:** The degree of perceived competencies among users influences the successful implementation of AI-based DSSs.	(+++)	In favor
**Reduction of uncertainties:** Due to reservations about AI-based DSSs, assessments could be categorically questioned by users, which can lead to DSSs not being used effectively.	(+++)	In favor
**Openness of potential users:** The openness of potential users towards AI-based DSSs is essential for implementation in hospitals.	(+++)	In favor
**Knowledge and understanding of how AI-based systems work:** It is important that human expertise is paired with the recommendations of AI-based DSSs (i.e., qualified people validate the results).	(+++)	In favor
**Perceived added value of the use of AI-based DSSs:** Only if users benefit noticeably from AI-based DSSs, can be implemented successfully in the long term.	(+++)	In favor
**Trust in the functioning of AI-based DSSs:** Trust of users in AI-based DSSs and its recommendations are essential for implementation.	(+++)	In favor
**Previous experience with AI-based DSSs:** Previous experience with AI-based DSSs is beneficial for successful implementation.	(++)	In favor
**Age of users:** The age of users influences the implementation and use of AI-based DSSs.^a^	(++)	In favor
**Professional experience:** The more professional experience users have, the greater the skepticism towards AI-based DSSs, which can have a negative impact on successful implementation.^a^	(x)	

Threshold for consensus in accordance with Meskell et al. (21):.

Consensus*:

“High” (+++) > >70% in category 4 or 3; >80% in categories 4 and 3.

“Moderate” (++) >60% in category 4 or 3; >70% in categories 4 and 3.

“Low” (+) >50% in category 4 or 3; >60% in categories 4 and 3.

“High” (—) >70% in category 2 or 1; >80% in categories 2 and 1.

“Moderate” (–) >60% in category 2 or 1; >70% in categories 2 and 1.

“Low” (-) >50% in category 2 or 1; >60% in categories 2 and 1.

“Dissent” (x): No unanimous group response.

N_1_ = 77; N_2_ = 36.

^a^ Results of the second round.

Though there is consensus on previous experience with AI-based DSSs to be beneficial for implementation, it must be considered how users perceive the experience:

“Previous experience with AI-based DSSs can hinder implementation if it [experience] was negative.” (Participant 2)

However, the experts remain in disagreement as to what extent the long-standing professional experience of (potential) users could be a hinderance to the implementation. Here 38.9% of the participants rather disagree and 8.3% disagree with the statement, that the more professional experience users have, the greater their skepticism towards AI-based DSSs might be, which can have a negative impact on successful implementation, while 19.4% of the participants agree and 30.6% rather agree.

### 3.3 Differences between clinical practice and research

The comparison of the professional role of clinical practice and research (*n* = 63 vs. *n* = 14) revealed differences in the assessment of the implementation factors (Additional File 1 Tables A1, A2). The mean value difference on the following factors are significant: manageable user interface with easy navigation (rather agree: 98.4% clinical practice vs. 78.6% research; *p* = 0.003); completeness of recommendations (agree: 76.2% clinical practice vs. 35.7% research; *p* = 0.021); assurance of technical support (agree: 87.3% clinical practice vs. 64.3% research; *p* = 0.018); promotion of the openness of potential users (agree: 74.6% clinical practice vs. 42.9% research; *p* = 0.018). The differences of the remaining implementation factors are not significant.

### 3.4 Ranking of implementation factors

To find the most significant factors influencing the implementation of AI-based DSSs for antibiotic prescribing in hospitals, Garrett's Ranking Technique is employed. To do this, the estimated percentage value was converted into point values using the Garrett table. The percentage score is calculated under the following formula (26):

$$\mathrm{Percentage}\;\mathrm{Score}=\frac{\;(\mathrm{Rij}\text{-}0,5)}{\mathrm{Nj}}$$

Where, R_ij_ = Rank given for i^th^ item j^th^ individual

N_j_ = Number of items ranked by j^th^ individual.

**Figure 3:**
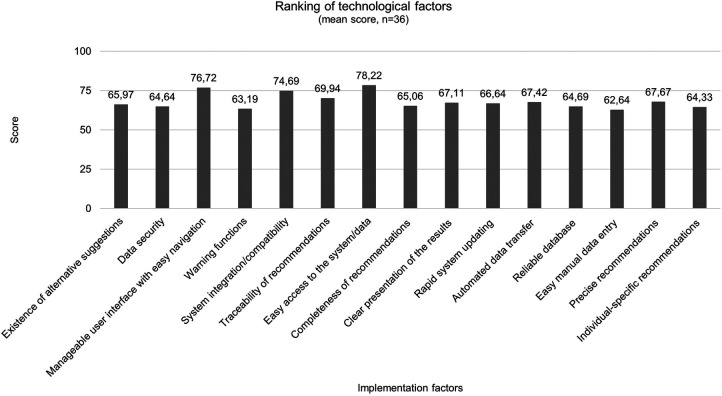
Ranking results of technological factors after second round (*n* = 36).

It is calculated as a percentage score (0–100) and the scale value is obtained by employing the Scale Conversion Table given by Henry Garrett (26). The higher the average score, the more important the factor under consideration (26). It is clear from Figure 3, that in the domain of technology the participants have given importance to the factors (the mean score is indicated in the brackets) system access (78.22), user interface (76.72), and system integration in existing technical structures (74.69).

**Figure 4:**
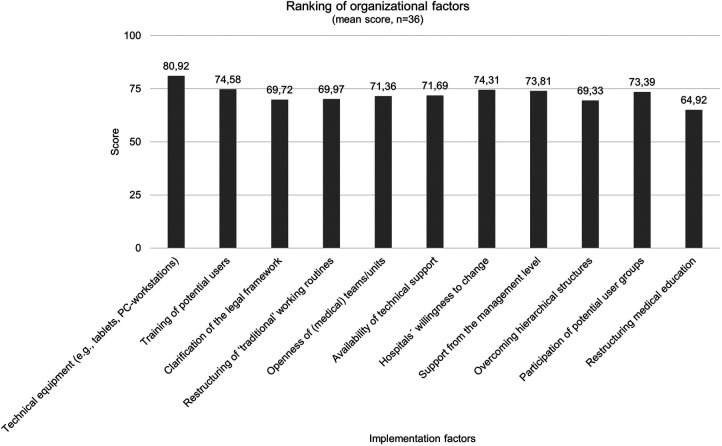
Ranking results of organizational factors after second round (*n* = 36).

Figure 4 shows the ranking results of organizational implementation factors that have reached consensus after the first round of the survey. Ensuring technical equipment is occupied the rank 1 (80.92), training offers for (potential) users occupied the rank 2 (74.58) and hospitalś willingness to change and integrate new technologies occupied the rank 3 (74.31). Restructuring of medical education (64.92) occupied the lowest rank in the table.

**Figure 5:**
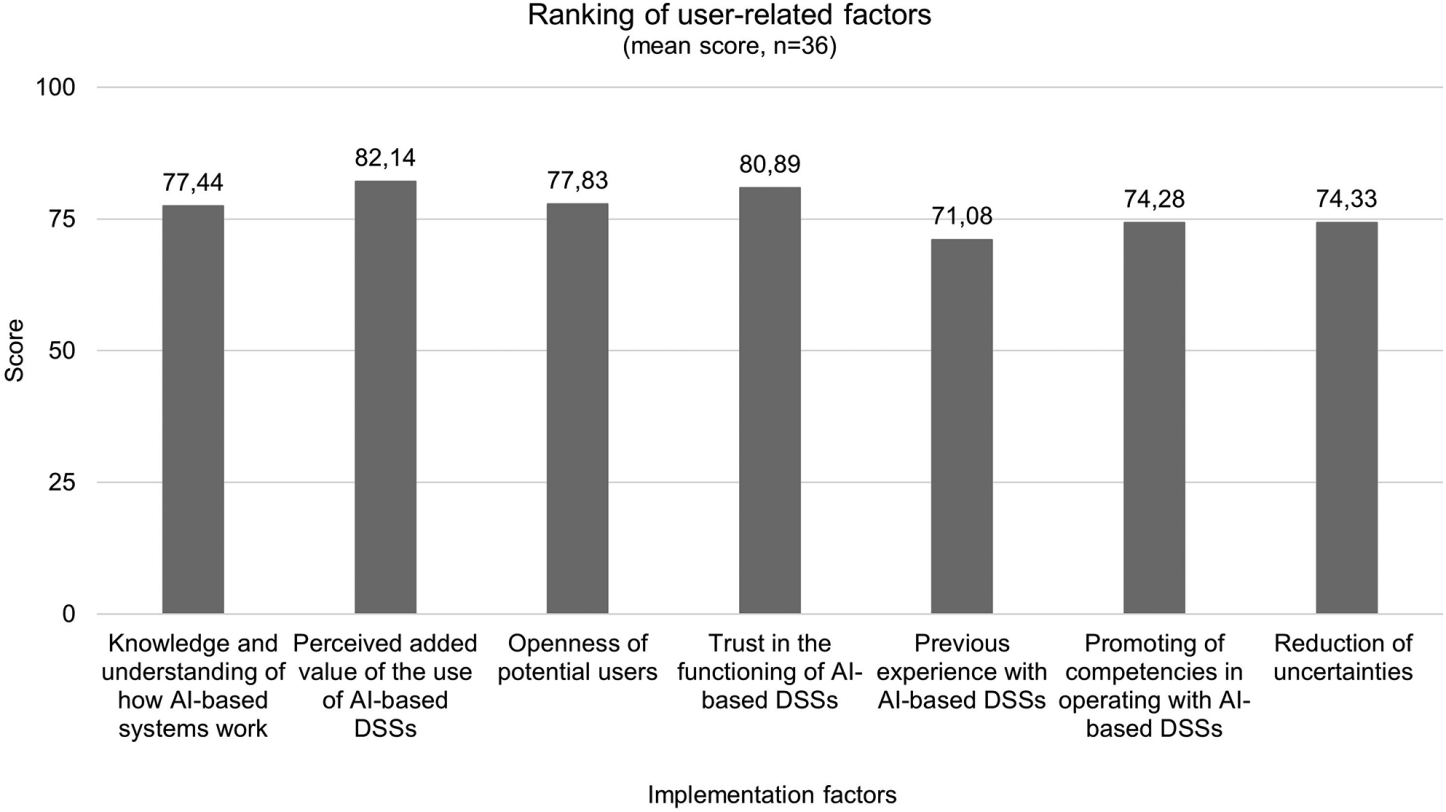
Ranking results of user-related factors after second round (*n* = 36).

With a view to the ranking of user-related factors that have reached consensus after the first round of the survey, it is evident that the perceived added value of AI-based DSSs (82.14) is the most important implementation factor, followed by trust in its functioning (80.89) and the openness of users to operate with it (77.83). With an average Garret score of 71.08 previous experience with AI-based DSSs is the least (Figure 5).

The details of the ranking of factors influencing the implementation of AI-based DSSs frequencies of the Henry Garret Ranking method are obtained in supplementary material (Additional File 2 Tables A1–A10).

### 3.5 Current realization of factors

The respondents were then asked for their assessment of the current realization of the factors that reached consensus in the first round. The results show that the participants mostly were unable to give a clear assessment of implementation. For example, eight respondents consider the factor that hospitals are supported in their willingness to change to be rather well realized, while six respondents rate this factor as not yet realized at all, and 15 respondents are unable to assess in this regard. In addition, ten participants rate ensuring data security as very good or rather good, while three participants rate it as rather not well realized and 23 could give no appraisal. Moreover, 13 respondents assess the sharing of knowledge in the context of AI-based DSSs as rather poorly or poorly realized, while nine respondents consider this factor as not realized at all, and 12 respondents could not give an assessment. The details of the appraisal of the current implementation of factors can be gathered in Table A1 in Additional File 3.

When asked with which interventions and strategies implementation might be promoted, one participant states, that developers should offer open interfaces that are freely available to every institution, instead of every development team having and providing their own interfaces, licenses, and databases, that cannot be acquired and used. Besides, the majority of AI-based DSSs that are currently being developed as prototypes by researchers are not comprehensible. More research in the field of explainable AI (XAI) could be a favorable factor in terms of traceability. In this context, in the development phase revision loops with a strong emphasis on usability evaluation should be pursued as well as the definition and effective application of interoperability standards. Moreover, the definition of a legal framework would be lagging behind the technical development, so it appears to be important for the state to be more active and to invest in the development of a cornerstone for the implementation and use of AI-based DSSs- meaning the technical and legal infrastructure and also to invest in the improvement of the digitalization level of hospitals, that are not operating with such systems, yet. In addition, the adaptation of the medical training should be envisaged as well as the establishment of multidisciplinary teams that would have been mandated with the support and monitoring of the implementation. Finally, raising awareness among (potential) users should be taken seriously to break the distorted facts about the use of AI and to report objectively on the possibilities and limitations of its use. This could be realized through e.g., workshops or technology showrooms.

### 3.6 Factors impacting trust in AI-based DSSs

**Figure 6:**
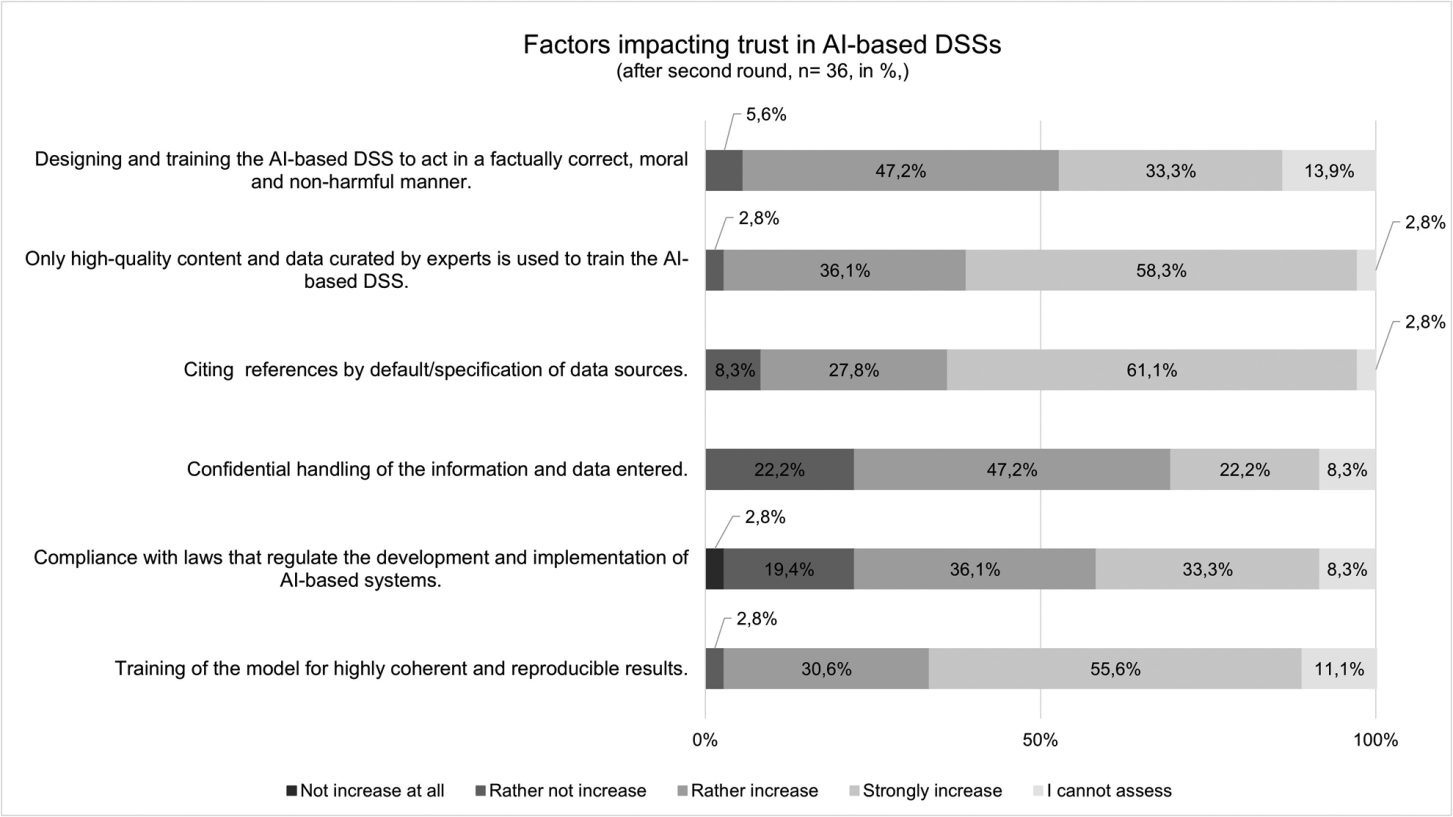
Assessment of factors influencing trust in AI-based DSSs (*n* = 36).

Respondents to the current survey share their views about how to build trust in AI-based DSSs (Figure 6). Almost half of the respondents say training AI-based DSSs to be factually accurate, moral, and not harmful would rather increase and more than a third of the respondents say would strongly increase their trust in that system. Additionally, almost 60% of respondents say only using high-quality content to train DSSs would strongly increase their trust, while 55% say training the model for highly coherent and reproducible results would strongly increase their trust. Transparency is also an important factor so for more than 60% of the respondents, citing references by default will strongly increase trust. The confidential handling of the information is rather a trust-facilitating factor for 47% and strongly facilitating for 22% of the respondents. More than a third of the respondents assessed compliance with laws as strongly increasing their trust.

In conclusion, looking to the future, 75% of the respondents (*n* = 27) agree that AI-based DSSs for antibiotic prescribing in hospitals will gain significance, while 25% consider them to gain slight importance.

## 4 Discussion

This study aimed to systematically evaluate AI-based DSSs and the factors that could potentially influence their implementation for antibiotic prescribing in hospitals from the perspective of a Delphi panel. The experts recognize the increasing importance of corresponding systems. The participants expect AI-based DSSs to be more present and incorporated into daily work routines in the future. The theses presented in the study can be combined to paint an overall picture of how AI-based DSSs might be incorporated into care and research in the future. The participants assess many of the arguments relating to the potential of AI-based DSSs positively. The experts see an added value in AI-based DSSs when it comes to providing guidance for antibiotic prescription as well as increasing treatment safety and improving daily work. Nevertheless, it has to be taken into account that adopting a supposedly correct result without proper consideration might provide the potential for medication errors (27). Even though DSSs facilitate decision-making and have the potential to reduce the workload, the users’ ability to act may be limited, when the system fails and the degree of reliance on the recommendations can generate a form of dependency as well (28). It is clear that the decision-making process of clinicians cannot be completely replaced by AI-based DSSs, nor that this would be desired by those involved (17, 29). Here it is necessary to clarify the tasks of AI-based DSSs in a participatory process and enable a continuous process of implementation.

Consensus has been established for 34 implementation factors: 15 technological factors, 11 organizational, and eight user-related factors. The respondents have then given their preferential ordering of the unranked implementation factors that reached a consensus at the initial stage. Garret´s Ranking Technique revealed that the most important factor influencing implementation in the domain of organization was the assurance of technical equipment with a mean score of 80.92. This also contains hospitalś willingness to incorporate innovations, which reached rank three in this study, and also to integrate responsible authorities, like the management level in this process to establish organizational structures for user-friendly implementation (30). When presented with the issue of “clarification of the legal framework”, the panel is in agreement to achieve this objective. The progress of AI in healthcare has consequences for the law. The legal challenges are also based on the risks associated with the systems, such as unpredictability and uncontrollability (31). From a legal perspective, the diffusion of responsibility, the relationship between healthcare professionals and patients as well as regulations concerning personal data are relevant topics (32). The protection of personal data is regulated in detail by the General Data Protection Regulation (GDPR) (33). Nevertheless, there is still a need for further discussion in context of AI-based systems. For example, the right to erasure under Art. 17 GDPR poses considerable difficulties, since the complete deletion of personal data that has already been processed from an AI-based system is technically difficult due to its technical architecture (34). An adaptation to the architecture of AI should therefore continue to be discussed. Concepts such as dynamic consent, the introduction of data trustees and the possibility of data donation enable the people to have more control over their data (35). Another aspect that is being discussed concerns the allocation of data as a type of special property. In this context, the question would arise as the system may use the data for further learning or whether it may be given to third parties in anonymized form, e. g., for further development of the system (36). Consequently, fundamental changes will be required, since there is also a lack of clarity concerning what authority and responsibility the users must assume. There are uncertainties regarding the distribution of roles and responsibilities that might be intensified by the use of AI-based DSSs, especially dealing with the recommendations as well as their binding nature, which possibly reduce the acceptance of the system (37). To address this, solutions for an appropriate distribution of responsibility are needed, whereby all relevant parties like developers, clinicians as (potential) users, hospitals, and their managers and experts for data protection and ethics (33). More attention has also to be paid to further education and training of medical staff in particular dealing with AI-based systems and the provision of necessary information. Linked with that, there is consensus among the experts that knowledge and competencies will be key factors in the future. A consensus study of a working group on AI in healthcare also concluded, that educators in healthcare should define new competencies for using AI-based DSS and these requirements should be incorporated into medical education (38). In this context, it is about digital literacy, e.g., the competent handling of the system and execution of the recommendations in practice, which has so far also lacking in medical education (39), even if this factor has been ranked the least important among the experts of this Delphi study. Moreover, the factor “perceived added value of AI-based DSSs”, has been ranked as most important in the user-related domain. In the context of evidence-based medicine, the safety, appropriateness, and efficacy of new interventions must be proven before they can be used in clinical practice. Following this paradigm, AI-based DSSs should also have an additional benefit before they can become part of routine care and be fully accepted by clinicians. Additionally, userś trust in DSSs has been ranked as the second important factor influencing implementation, which is in line with previous studies (40, 41). Trust can be achieved by creating transparency regarding the extent to which the recommendations of DSSs apply to the context in which they are embedded in a value-oriented manner (42). Explainability is but one indicator of achieving transparency as well as an open communication on the systemś capabilities and limitations (43). Methods from the field of XAI are important here. The so-called global explanation describes which variables are particularly relevant for the model in general and how strongly they influence the systemś recommendations (44). However, a specific decision can also be based partly on other factors - this is referred to as local explanation (45). Although neither of these factors makes it possible to open the “black box”, together with information on the training data, the methodology used and the results of validation studies, it increases the transparency of the system and gives users an indication of how it works internally, which can promote trust in DSSs (46). In this issue, a study analyzing the development and implementation of a DSS for antimicrobial stewardship in two Swiss hospitals highlights the fact, that making the underlying process of decision-making transparent and understandable for clinicians they were more willing to accept the system (47). In addition, it was beneficial to keep the process simple and visible for users as well as make clear for them where the recommendations, respective the content is coming from (47). Not all users need to understand the system as a whole to trust it, but they should know why they can trust it (48). This is also reflected in the respondents' assessments of factors that influence trust in DSSs. Here, the data and evidence on which the recommendations are based play significant roles for the majority of respondents. However, evidence-based medicine does not only refer to the effectiveness of the respective systems as determined by scientific standards but also to linking this to the expertise of medical staff in the best possible way (49). Concerning technological factors “easy access to the system/data” reached the first rank with a mean score of 78.22. This is in line with the results of another Delphi study, where consensus was sought on a core list of important safety features to be considered when designing, implementing or using DSSs (50). The panelists agreed on items related to the ease of using the system and also that the system should provide prompts to specifying doses of the medications prescribed, frequencies and dosage forms (50), which also reached consensus in this Delphi study.

At this point, it seems to be important to figure out what is technically possible and how the technical possibilities can be utilized most profitably. Generally speaking, DSSs should be designed along with clinical needs and not only according to the developerś possibilities. The fit of the technology with clinical processes was also addressed in a study in which the integration of a DSS in an Austrian hospital was described (51). Here it proved to be a challenge that those developing the technology were not familiar with the organizational processes. Therefore, implementation failed and could only be continued after appropriate adjustments. It also became apparent that the lack of cooperation with clinical stakeholders meant that the added value of DSS for improving quality of care and working routines was not recognized or accepted (51). In addition, it appears that system attributes of AI-based DSSs have to simultaneously meet the demands of e.g., easy access, compatibility, transparency, or explainability, whereby not everything can be optimized at the same time. One opportunity to apply to this demands is to provide key facts for interested and informed users in the form of an easy-to-understand manual, so-called model cards, about the underlying model, characteristics of the training data, and system evaluation (52). Furthermore, the requirement to make the recommendations of an AI-based DSS more explainable will make it necessary to involve medical staff more in the development of such systems (53), which also reached a consensus within the Delphi panel. It is also important to consider the items that did not reach consensus. In this context, there is no clear tendency relating professional experience as being a hinderance or facilitator for DSS implementation. It might be possible that the sample composition had an influence on the results. In this study, people participated, who are clinical experts and just in the beginning of their professional career and probably more open towards innovative ways of care, as people who have more professional experience and are more reserved towards change. It is well known that in deep-rooted systems and hierarchies characterized by seniority like in hospitals, there might be resistance towards scientific evidence (54). Besides, expertise have also been found to influence perceptions and use of DSS implementation (55), so that the heterogeneity of expert levels in this study have also affected the assessment of implementation factors.

However, as it is apparent from the appraisal of the current realization of the factors if AI-based DSSs for antibiotic prescribing are to be considered reliable, safe, and beneficial to human well-being, comprehensive strategies and frameworks for their use in the healthcare sector will need to be developed over the next few years in collaboration with various stakeholders.

## 5 Strengths and limitations

This study was the first to systematically determine which factors should be considered when implementing AI-based DSSs in hospital settings from the perspective of experts in the field of antibiotic prescribing as well as AI-based DSSs in practice and research.

Even though the methodology of the Delphi method is an accepted procedure in the scientific community, the results reflect an accumulation of subjective judgements. There is always a degree of uncertainty about current and future developments. The appraisal of influencing factors is based on the expectations of the sample and is not representative (56). The challenging identification and recruitment of experts (especially in the field of development and deployment of AI-based DSSs for the hospital setting) indicates that there are still few specialists in this area. In addition, the survey results largely reflect the assessment of ASP-members in hospitals, as these are overrepresented in the survey. The overrepresentation of clinical experts might have affected the prioritization of implementation factors, insofar that technical requirements were seen as less as a concern than organizational and user-related factors. A more balanced panel would be particularly desirable in the context of the variability of perspectives. Further on, one conclusion from the response rates of 15% in the first and 47% in the second round is that on the one hand, the two-step process may have been too time-consuming and on the other hand the questionnaire was possibly too extensive. However, this seemed necessary to gain an overview of the manifold factors influencing implementation in healthcare facilities like hospitals. For further research, a shorter and more focused questionnaire should be developed. Still, the panel size was within the range of sizes used in previous studies involving achieving consensus on issues in healthcare (57, 58). The high level of expertise of the sample concerning context-related antibiotic prescription speaks in favor of high informative value. The rather moderate to low expertise in the field of DSSs might be a possible reason for the inconclusive assessment of current realization of implementation factors, since existing knowledge gaps as well as insufficient implementation strategies and activities in German hospitals may have had an impact on the assessment, which is limiting. Nonetheless, the results provide a broad overview from a context-related perspective about what seems to be important and relevant in terms of DSS implementation in hospital setting. That means that the results of the survey can be used to develop tools and frameworks that support the planning and implementation of AI-based DSSs in hospitals and the people involved.

## 6 Conclusion

With the aid of the Delphi method, it was possible to provide insights into factors related to the implementation of AI-based DSSs for antibiotic prescribing in German hospitals from the perspective of experts, whereby a wide range of factors seem to be crucial. Although there are promising examples of good practices for the use of AI-based DSSs in healthcare available, the extent of professional role-related or even overall societal effects are hardly foreseeable. Requirements of AI-based DSSs in everyday clinical practice are to promote application-orientated development as well as integrate them into clinical organizational structures and fulfill demands of trust, transparency, and responsibility. Given considerable uncertainties regarding the effects of AI-based DSS implementation in healthcare, the organizational, social, and ethical aspects of this transformation process must be given more attention than before, both in practice and research.

## Data Availability

The original contributions presented in the study are included in the article/Supplementary Material, further inquiries can be directed to the corresponding authors.
