# Supplementary material for: Implementation of artificial intelligence-based decision support systems for antibiotic prescribing in hospitals: a Delphi study
Source: Front Digit Health. 2025 Apr 25;7:1555042. doi: 10.3389/fdgth.2025.1555042 (PMC12062133; doi:10.3389/fdgth.2025.1555042)
Supplement: Supplementary file 1 [file Table1.docx]

Additional File 1

Table A1: Test statistics on group differences by professional role (Mann-Whitney-U-Test^a^) based on the results of the first round (n=77)

| **Implementation factor** | **Mann-Whitney-U-Test** | **Z^b^** | **Asymp. Sig. (two-sided)** |
| --- | --- | --- | --- |
| **Technological factors** | | | |
| Easy access to the system/ data | 378,000 | -1,282 | 0,200 |
| **Manageable user interface with easy navigation** | **353,500** | **-3,006** | **0,003*** |
| System integration/ compatibility | 402,000 | -0,848 | 0,397 |
| Traceability of recommendations | 388,000 | -0,971 | 0,331 |
| Precise recommendations | 424,500 | -0,241 | 0,809 |
| Automated data transfer | 416,500 | -0,497 | 0,619 |
| Clear presentation of the results | 399,000 | -1,300 | 0,194 |
| Rapid system updating | 372,500 | -1,629 | 0,104 |
| Existence of alternative suggestions | 324,500 | -1,867 | 0,062 |
| **Completeness of recommendations** | **299,000** | **-2,310** | **0,021*** |
| Reliable database | 416,500 | -1,174 | 0,240 |
| Data security | 336,000 | -1,664 | 0,096 |
| Individual-specific recommendations | 331,000 | -1,510 | 0,131 |
| Warning functions | 343,500 | -1,629 | 0,103 |
| Easy manual data entry | 354,000 | -1,232 | 0,218 |
| **Organizational factor** | | | |
| Promotion of the hospital's willingness to change | 349,500 | -1,607 | 0,108 |
| Restructuring of “traditional” working routines | 382,500 | -1,029 | 0,303 |
| Promotion of the openness of medical teams/units | 349,000 | -1,647 | 0,100 |
| Assurance of technical equipment | 396,000 | -0,864 | 0,388 |
| **Assurance of technical support** | **324,500** | **-2,366** | **0,018*** |
| Clarification of the legal framework | 378,000 | -1,495 | 0,135 |
| Restructuring medical education | 390,5000 | -0,762 | 0,446 |
| Assurance of support from the management level | 440,000 | -0,015 | 0,988 |
| Users’ participation in the development and implementation phase | 327,000 | -1,810 | 0,070 |
| Training of potential users | 430,500 | -0,189 | 0,850 |
| Overcoming hierarchical structures | 401,000 | -0,554 | 0,579 |
| Financial incentives | 422,000 | -0,259 | 0,795 |
| **User-related factors** | | | |
| Promotion of competencies in operating with AI-based DSSs | 421,500 | -0,277 | 0,782 |
| Reduction of uncertainties | 410,000 | -0,454 | 0,650 |
| **Promotion of the openness of potential users** | **297,000** | **-2,358** | **0,018*** |
| Sharing of knowledge and understanding of how AI-based systems work | 396,500 | -0,725 | 0,468 |
| Perceiving the added value of the use of AI-based DSSs | 413,000 | -0,504 | 0,614 |
| Promotion of trust in the functioning of AI-based DSSs | 438,000 | -0,065 | 0,948 |
| Professional experience | 364,000 | -1,057 | 0,290 |
| Age of users | 412,500 | -0,393 | 0,695 |
| Previous experience with AI-based DSSs | 398,000 | -0,597 | 0,550 |

^a^ Group variable: professional role binary (clinical practice & research)

^b^ The Z value indicates the strength of the difference between the two groups. The further the Z value is from zero, the greater the difference between the groups.

* significant result

Table A2: Group differences in relative frequencies (%) by professional role based on the results of the first round (n=77)

| **Factors** | **Professional role** | **Disagree** | **Rather disagree** | **Rather agree** | **Agree** | **I cannot assess** |
| --- | --- | --- | --- | --- | --- | --- |
| **Technological factors** | | | | | | |
| Easy access to the system/ data | Clinical practice (n=63) | - | - | 14,3% | 85,7% |  |
|  | Research (n=14) | - | - | 28,6% | 71,4% |  |
| **Manageable user interface with easy navigation** | Clinical practice (n=63) | - | - | **1,6%** | **98,4%** |  |
|  | Research (n=14) | - | - | **21,4%** | **78,6%** |  |
| System integration/ compatibility | Clinical practice (n=63) | - | - | 9,5% | 85,7% | 4,8% |
|  | Research (n=14) | - | - | 14,3% | 85,7% |  |
| Traceability of recommendations | Clinical practice (n=63) | - | - | 19,0% | 79,4% | 1,6% |
|  | Research (n=14) | 7,1% | - | 21,5% | 71,4% |  |
| Precise recommendations | Clinical practice (n=63) | 1,6% | 7,9% | 34,9% | 54,0% | 1,6% |
|  | Research (n=14) | - | 14,3% | 35,7% | 42,9% | 7,1% |
| Automated data transfer | Clinical practice (n=63) | - | - | 11,1% | 88,9% |  |
|  | Research (n=14) | - | 7,1% | 21,4% | 57,1% | 14,3% |
| Clear presentation of the results | Clinical practice (n=63) | - | - | 4,8% | 95,2% |  |
|  | Research (n=14) | - | - | 14,3% | 85,7% |  |
| Rapid system updating | Clinical practice (n=63) | - | - | 6,3% | 93,7% |  |
|  | Research (n=14) |  |  | 28,6% | 64,3% | 7,1% |
| Existence of alternative suggestions | Clinical practice (n=63) | 3,2% | 3,2% | 17,5% | 74,6% | 1,6% |
|  | Research (n=14) | - | 7,1% | 50,0% | 35,7% | 7,1% |
| **Completeness of recommendations** | Clinical practice (n=63) | **-** | **1,6%** | **22,2%** | **76,2%** |  |
|  | Research (n=14) | **7,1%** | **14,3%** | **35,7%** | **35,7%** | **7,1%** |
| Reliable database | Clinical practice (n=63) | - | - | 1,6% | 98,4% |  |
|  | Research (n=14) | - | - | 7,1% | 92,9% |  |
| Data security | Clinical practice (n=63) | 1,6% | 1,6% | 19,0% | 71,4% | 6,3% |
|  | Research (n=14) | - | - | 50,0% | 42,9% | 7,1% |
| Individual-specific recommendations | Clinical practice (n=63) | 3,2% | 27,0% | 30,2% | 34,9% | 4,8% |
|  | Research (n=14) | 21,4% | 28,6% | 28,6% | 7,1% | 14,3% |
| Warning functions | Clinical practice (n=63) | - | - | 19,0% | 76,2% | 4,8% |
|  | Research (n=14) | - | 7,1% | 35,7% | 50,0% | 7,1% |
| Easy manual data entry | Clinical practice (n=63) | 1,6% | 12,7% | 25,4% | 52,4% | 7,9% |
|  | Research (n=14) | 14,3% | 21,4% | 21,4% | 28,6% | 14,3% |
| **Organizational factors** | | | | | | |
| Promotion of the hospital's willingness to change | Clinical practice (n=63) | - | 1,6% | 15,9% | 77,8% | 4,8% |
|  | Research (n=14) | - | - | 35,7% | 64,3% | - |
| Restructuring of “traditional” working routines | Clinical practice (n=63) | - | - | 19,0% | 79,4% | 1,6% |
|  | Research (n=14) | 7,1% | - | 28,6% | 57,1% | 7,1% |
| Promotion of the openness of medical teams/units | Clinical practice (n=63) | - | 3,2% | 20,6% | 74,6% | 1,6% |
|  | Research (n=14) | - | - | 7,1% | 85,7% | 7,1% |
| Assurance of technical equipment | Clinical practice (n=63) | - | 3,2% | 14,3% | 82,5% | - |
|  | Research (n=14) | - | - | 28,6% | 71,4% | - |
| **Assurance of technical support** | Clinical practice (n=63) | - | **-** | **11,1%** | **87,3%** | **1,6%** |
|  | Research (n=14) | - | **7,1%** | **28,6%** | **64,3%** | **-** |
| Clarification of the legal framework | Clinical practice (n=63) | - | - | 14,3% | 85,7% | - |
|  | Research (n=14) | - | - | - | 100,0% | - |
| Restructuring medical education | Clinical practice (n=63) | - | 7,9% | 30,2% | 60,3% | 1,6% |
|  | Research (n=14) | - | 7,1% | 42,9% | 50,0% | - |
| Assurance of support from the management level | Clinical practice (n=63) | - | 3,2% | 31,7% | 65,1% | - |
|  | Research (n=14) | - | 7,1% | 35,7% | 42,9% | 14,3% |
| Users’ participation in the development and implementation phase | Clinical practice (n=63) | - | 3,2% | 36,5% | 60,3% | - |
|  | Research (n=14) | - | - | 14,3% | 85,7% | - |
| Training of potential users | Clinical practice (n=63) | - | - | 23,8% | 76,2% | - |
|  | Research (n=14) | - | - | 21,4% | 78,6% | - |
| Overcoming hierarchical structures | Clinical practice (n=63) | 1,6% | 25,4% | 39,7% | 30,2% | 3,2% |
|  | Research (n=14) | 7,1% | 35,7% | 28,6% | 7,1% | 21,4% |
| Financial incentives | Clinical practice (n=63) | 11,1% | 36,5% | 27,0% | 17,5% | 7,9% |
|  | Research (n=14) | 21,4% | 21,4% | 21,4% | 21,4% | 14,3% |
| **User-related factors** | | | | | | |
| Promotion of competencies in operating with AI-based DSSs | Clinical practice (n=63) | - | 4,8% | 36,5% | 50,8% | 7,9% |
|  | Research (n=14) | 7,1% | 7,2% | 35,7% | 14,3% | 35,7% |
| Reduction of uncertainties | Clinical practice (n=63) | - | 9,5% | 36,5% | 50,8% | 3,2% |
|  | Research (n=14) | - | - | 57,1% | 42,9% | - |
| **Promotion of the openness of potential users** | Clinical practice (n=63) | - | **1,6%** | **23,8%** | **74,6%** | - |
|  | Research (n=14) | - | **7,1%** | **50,0%** | **42,9%** | - |
| Sharing of knowledge and understanding of how AI-based systems work | Clinical practice (n=63) | - | 1,6% | 27,0% | 68,3% | 3,2% |
|  | Research (n=14) | - | - | 21,4% | 71,4% | 7,1% |
| Showing the added value of the use of AI-based DSSs | Clinical practice (n=63) | - | - | 22,2% | 77,8% | - |
|  | Research (n=14) | - | - | 28,6% | 71,4% | - |
| Promotion of trust in the functioning of AI-based DSSs | Clinical practice (n=63) | 1,6% | - | 12,7% | 85,7% | - |
|  | Research (n=14) | - | 7,1% | 7,1% | 85,7% | - |
| Professional experience | Clinical practice (n=63) | 9,5% | 39,7% | 28,6% | 14,3% | 7,9% |
|  | Research (n=14) | 35,7% | 28,6% | 7,1% | 7,1% | 21,4% |
| Age of users | Clinical practice (n=63) | 14,3% | 22,2% | 41,3% | 15,9% | 6,3% |
|  | Research (n=14) | 21,4% | 14,3% | 35,7% | - | 28,6% |
| Facilitating experience with AI-based DSSs before actual use | Clinical practice (n=63) | 1,6% | 17,5% | 41,3% | 31,7% | 7,9% |
|  | Research (n=14) | 21,4% | 7,1% | 35,7% | 21,4% | 14,3% |
