# Supplementary material for: Implementation of artificial intelligence-based decision support systems for antibiotic prescribing in hospitals: a Delphi study
Source: Front Digit Health. 2025 Apr 25;7:1555042. doi: 10.3389/fdgth.2025.1555042 (PMC12062133; doi:10.3389/fdgth.2025.1555042)
Supplement: Supplementary file 2 [file Table2.docx]

Additional file 2

Table A1: Ranks given for technological factors by the respondents (n=36)

| **Factors** | **Rank 1** | **Rank 2** | **Rank 3** | **Rank 4** | **Rank 5** | **Rank 6** | **Rank 7** | **Rank 8** | **Rank 9** | **Rank 10** | **Rank 11** | **Rank 12** | **Rank 13** | **Rank 14** | **Rank 15** |
| --- | --- | --- | --- | --- | --- | --- | --- | --- | --- | --- | --- | --- | --- | --- | --- |
| Existence of alternative suggestions | 2 | 1 | 2 | 0 | 4 | 2 | 3 | 4 | 1 | 2 | 4 | 2 | 3 | 3 | 3 |
| Data security | 4 | 2 | 2 | 0 | 1 | 0 | 1 | 2 | 1 | 0 | 4 | 1 | 4 | 4 | 10 |
| Manageable user interface with easy navigation | 7 | 4 | 6 | 5 | 2 | 3 | 4 | 3 | 1 | 0 | 0 | 0 | 0 | 1 | 0 |
| Warning functions | 0 | 1 | 1 | 1 | 0 | 3 | 1 | 3 | 5 | 5 | 3 | 5 | 4 | 1 | 3 |
| System integration/ compatibility | 6 | 7 | 2 | 5 | 5 | 0 | 1 | 0 | 3 | 1 | 0 | 3 | 2 | 0 | 1 |
| Traceability of recommendations | 4 | 4 | 2 | 1 | 0 | 2 | 3 | 3 | 5 | 5 | 2 | 3 | 1 | 1 | 0 |
| Easy access to the system/ data | 11 | 6 | 3 | 2 | 4 | 3 | 1 | 1 | 0 | 1 | 2 | 0 | 1 | 1 | 0 |
| Completeness of recommendations | 0 | 1 | 2 | 2 | 2 | 3 | 1 | 5 | 6 | 3 | 1 | 1 | 5 | 1 | 3 |
| Clear presentation of the results | 0 | 0 | 3 | 2 | 5 | 4 | 5 | 1 | 4 | 5 | 3 | 2 | 0 | 1 | 1 |
| Rapid system updating | 0 | 1 | 2 | 6 | 6 | 1 | 2 | 1 | 1 | 3 | 4 | 0 | 4 | 4 | 1 |
| Automated data transfer | 0 | 4 | 2 | 4 | 2 | 4 | 2 | 2 | 2 | 1 | 3 | 4 | 1 | 3 | 2 |
| Reliable database | 1 | 0 | 3 | 0 | 3 | 3 | 3 | 2 | 3 | 2 | 2 | 5 | 2 | 5 | 2 |
| Easy manual data entry | 0 | 0 | 1 | 2 | 1 | 3 | 3 | 1 | 1 | 2 | 6 | 7 | 4 | 2 | 3 |
| Precise recommendations | 1 | 4 | 1 | 4 | 0 | 4 | 4 | 6 | 0 | 1 | 1 | 2 | 0 | 4 | 4 |
| Individual-specific recommendations | 0 | 1 | 4 | 2 | 1 | 1 | 2 | 2 | 3 | 5 | 1 | 1 | 5 | 5 | 3 |

Table A2: Percent positions and Garret value of technological factors

| **Rank** | **100(Rij-0,5)/N** | **Percent position** | **Garret value** |
| --- | --- | --- | --- |
| 1 | 100(1-0,5)/36 | 1,39 | 91 |
| 2 | 100(2-0,5)/36 | 4,17 | 83 |
| 3 | 100(3-0,5)/36 | 6,94 | 79 |
| 4 | 100(4-0,5)/36 | 9,72 | 75 |
| 5 | 100(5-0,5)/36 | 12,50 | 72 |
| 6 | 100(6-0,5)/36 | 15,28 | 70 |
| 7 | 100(7-0,5)/36 | 18,06 | 68 |
| 8 | 100(8-0,5)/36 | 20,83 | 66 |
| 9 | 100(9-0,5)/36 | 23,61 | 64 |
| 10 | 100(10-0,5)/36 | 26,39 | 63 |
| 11 | 100(11-0,5)/36 | 29,17 | 61 |
| 12 | 100(12-0,5)/36 | 31,94 | 59 |
| 13 | 100(13-0,5)/36 | 34,72 | 57 |
| 14 | 100(14-0,5)/36 | 37,50 | 56 |
| 15 | 100(15-0,5)/36 | 40,28 | 55 |

Table A3: The Calculation of Garret score and ranking of technological factors

| **Factors** | **Rank 1* 91** | **Rank 2*83** | **Rank 3*79** | **Rank 4*75** | **Rank 5*72** | **Rank 6*70** | **Rank 7*68** | **Rank 8*66** | **Rank 9*64** | **Rank 10*63** | **Rank 11*61** | **Rank 12*59** | **Rank 13*57** | **Rank 14*56** | **Rank 15*55** | **Total** |
| --- | --- | --- | --- | --- | --- | --- | --- | --- | --- | --- | --- | --- | --- | --- | --- | --- |
| Existence of alternative suggestions | 182 | 83 | 158 | 0 | 288 | 140 | 204 | 364 | 64 | 126 | 244 | 118 | 171 | 168 | 165 | 2375 |
| Data security | 364 | 166 | 158 | 0 | 72 | 0 | 68 | 132 | 64 | 0 | 244 | 59 | 228 | 224 | 550 | 2329 |
| Manageable user interface with easy navigation | 637 | 332 | 474 | 375 | 144 | 210 | 272 | 198 | 64 | 0 | 0 | 0 | 0 | 56 | 0 | 2762 |
| Warning functions | 0 | 83 | 79 | 75 | 0 | 210 | 68 | 198 | 320 | 315 | 183 | 295 | 228 | 56 | 165 | 2275 |
| System integration/ compatibility | 546 | 581 | 158 | 375 | 360 | 0 | 68 | 0 | 192 | 63 | 0 | 177 | 114 | 0 | 55 | 2689 |
| Traceability of recommendations | 364 | 332 | 158 | 75 | 0 | 140 | 204 | 198 | 320 | 315 | 122 | 177 | 57 | 56 | 0 | 2518 |
| Easy access to the system/ data | 1001 | 498 | 237 | 150 | 288 | 210 | 68 | 66 | 0 | 63 | 122 | 0 | 57 | 56 | 0 | 2816 |
| Completeness of recommendations | 0 | 83 | 158 | 150 | 144 | 210 | 68 | 330 | 384 | 189 | 61 | 59 | 285 | 56 | 165 | 2342 |
| Clear presentation of the results | 0 | 0 | 237 | 150 | 360 | 280 | 340 | 66 | 259 | 315 | 183 | 118 | 0 | 56 | 55 | 2416 |
| Rapid system updating | 0 | 83 | 158 | 450 | 432 | 70 | 136 | 66 | 64 | 189 | 244 | 0 | 228 | 224 | 55 | 2399 |
| Automated data transfer | 0 | 332 | 158 | 300 | 144 | 280 | 136 | 132 | 128 | 63 | 183 | 236 | 57 | 168 | 110 | 2427 |
| Reliable database | 91 | 0 | 237 | 0 | 261 | 210 | 204 | 132 | 192 | 126 | 122 | 295 | 114 | 280 | 110 | 2329 |
| Easy manual data entry | 0 | 0 | 79 | 150 | 72 | 210 | 204 | 66 | 64 | 126 | 366 | 413 | 228 | 112 | 165 | 2255 |
| Precise recommendations | 91 | 332 | 79 | 300 | 0 | 280 | 272 | 396 | 0 | 63 | 61 | 118 | 0 | 224 | 220 | 2436 |
| Individual-specific recommendations | 0 | 83 | 316 | 150 | 72 | 70 | 136 | 132 | 192 | 315 | 61 | 59 | 285 | 280 | 165 | 2316 |

Table A4: Calculation of Garret score and ranking of technological factors (continuation)

| **Factors** | **Total** | **Score**  **(Total/N)** | **Ranking** |
| --- | --- | --- | --- |
| Existence of alternative suggestions | 2375 | 65,97 | 9 |
| Data security | 2329 | 64,64 | 12 |
| Manageable user interface with easy navigation | 2762 | 76,72 | 2 |
| Warning functions | 2275 | 63,19 | 14 |
| System integration/ compatibility | 2689 | 74,69 | 3 |
| Traceability of recommendations | 2518 | 69,94 | 4 |
| Easy access to the system/ data | 2816 | 78,22 | 1 |
| Completeness of recommendations | 2342 | 65,06 | 10 |
| Clear presentation of the results | 2416 | 67,11 | 7 |
| Rapid system updating | 2399 | 66,64 | 8 |
| Automated data transfer | 2427 | 67,42 | 6 |
| Reliable database | 2329 | 64,69 | 11 |
| Easy manual data entry | 2255 | 62,64 | 15 |
| Precise recommendations | 2436 | 67,67 | 5 |
| Individual-specific recommendations | 2316 | 64,33 | 13 |

| **Factors** | **Rank 1** | **Rank 2** | **Rank 3** | **Rank 4** | **Rank 5** | **Rank 6** | **Rank 7** | **Rank 8** | **Rank 9** | **Rank 10** | **Rank 11** |
| --- | --- | --- | --- | --- | --- | --- | --- | --- | --- | --- | --- |
| Technical equipment (e.g., tablets, PC-workstations) | 11 | 10 | 5 | 1 | 2 | 2 | 1 | 2 | 2 | 0 | 0 |
| Training of potential users | 5 | 4 | 5 | 4 | 4 | 0 | 3 | 5 | 3 | 3 | 0 |
| Clarification of the legal framework | 4 | 3 | 0 | 2 | 2 | 1 | 5 | 2 | 6 | 3 | 8 |
| Restructuring of “traditional” working routines | 0 | 2 | 2 | 3 | 1 | 6 | 3 | 4 | 3 | 7 | 5 |
| Openness of (medical) teams/units | 0 | 3 | 4 | 2 | 5 | 10 | 5 | 3 | 4 | 0 | 0 |
| Availability of technical support | 1 | 5 | 5 | 5 | 3 | 0 | 3 | 2 | 3 | 6 | 3 |
| Hospitals´ willingness to change | 7 | 2 | 1 | 4 | 5 | 4 | 2 | 8 | 2 | 0 | 1 |
| Support from the management level | 4 | 2 | 5 | 6 | 6 | 2 | 2 | 2 | 5 | 0 | 2 |
| Overcoming hierarchical structures | 1 | 2 | 2 | 3 | 3 | 4 | 7 | 3 | 1 | 6 | 4 |
| Participation of potential user groups in the development and implementation phase | 3 | 3 | 7 | 5 | 2 | 3 | 2 | 3 | 2 | 5 | 1 |
| Restructuring medical education | 0 | 0 | 0 | 1 | 3 | 4 | 3 | 2 | 5 | 6 | 12 |

Table A5: Ranks given for organizational factors by the respondents (n=36)

Table A6: Percent positions and Garret value of organizational factors

| **Rank** | **100(Rij-0,5)/N** | **Percent position** | **Garret value** |
| --- | --- | --- | --- |
| 1 | 100(1-0,5)/36 | 1,39 | 91 |
| 2 | 100(2-0,5)/36 | 4,17 | 83 |
| 3 | 100(3-0,5)/36 | 6,94 | 79 |
| 4 | 100(4-0,5)/36 | 9,72 | 75 |
| 5 | 100(5-0,5)/36 | 12,50 | 72 |
| 6 | 100(6-0,5)/36 | 15,28 | 70 |
| 7 | 100(7-0,5)/36 | 18,06 | 68 |
| 8 | 100(8-0,5)/36 | 20,83 | 66 |
| 9 | 100(9-0,5)/36 | 23,61 | 64 |
| 10 | 100(10-0,5)/36 | 26,39 | 63 |
| 11 | 100(11-0,5)/36 | 29,17 | 61 |

Table A7: Calculation of Garret score and ranking of organizational factors

| **Factors** | **Rank 1* 91** | **Rank 2*83** | **Rank 3*79** | **Rank 4*75** | **Rank 5*72** | **Rank 6*70** | **Rank 7*68** | **Rank 8*66** | **Rank 9*64** | **Rank 10*63** | **Rank 11*61** | **Total** | **Score (Total/N)** | **Ranking** |
| --- | --- | --- | --- | --- | --- | --- | --- | --- | --- | --- | --- | --- | --- | --- |
| Technical equipment (e.g., tablets, PC-workstations) | 1001 | 830 | 395 | 75 | 144 | 140 | 68 | 132 | 128 | 0 | 0 | 2913 | 80,92 | 1 |
| Training of potential users | 455 | 332 | 395 | 300 | 288 | 0 | 204 | 330 | 192 | 189 | 0 | 2685 | 74,58 | 2 |
| Clarification of the legal framework | 364 | 249 | 0 | 150 | 144 | 70 | 340 | 132 | 384 | 189 | 488 | 2510 | 69,72 | 9 |
| Restructuring of “traditional” working routines | 0 | 166 | 158 | 225 | 72 | 420 | 204 | 264 | 192 | 441 | 305 | 2447 | 69,97 | 8 |
| Openness of (medical) teams/units | 0 | 249 | 316 | 150 | 360 | 700 | 340 | 198 | 256 | 0 | 0 | 2569 | 71,36 | 7 |
| Availability of technical support | 91 | 415 | 395 | 375 | 216 | 0 | 204 | 132 | 192 | 378 | 183 | 2581 | 71,69 | 6 |
| Hospitals´ willingness to change | 637 | 166 | 79 | 300 | 360 | 280 | 136 | 528 | 128 | 0 | 61 | 2675 | 74,31 | 3 |
| Support from the management level | 364 | 166 | 395 | 450 | 432 | 140 | 136 | 132 | 320 | 0 | 122 | 2657 | 73,81 | 4 |
| Overcoming hierarchical structures | 91 | 166 | 158 | 225 | 216 | 280 | 476 | 198 | 64 | 378 | 244 | 2496 | 69,33 | 10 |
| Participation of potential user groups in the development and implementation phase | 273 | 249 | 553 | 375 | 144 | 210 | 136 | 198 | 128 | 315 | 61 | 2642 | 73,39 | 5 |
| Restructuring medical education | 0 | 0 | 0 | 75 | 216 | 280 | 204 | 132 | 320 | 378 | 732 | 2337 | 64,92 | 11 |

Table A8: Ranks given for user-related factors by the respondents (n=36)

| **Factors** | **Rank 1** | **Rank 2** | **Rank 3** | **Rank 4** | **Rank 5** | **Rank 6** | **Rank 7** |
| --- | --- | --- | --- | --- | --- | --- | --- |
| Knowledge and understanding of how AI-based systems work | 7 | 3 | 5 | 7 | 5 | 5 | 4 |
| Perceived added value of the use of AI-based DSSs | 14 | 5 | 6 | 4 | 4 | 1 | 2 |
| Openness of potential users | 7 | 6 | 3 | 6 | 5 | 4 | 5 |
| Trust in the functioning of AI-based DSSs | 8 | 11 | 7 | 2 | 4 | 4 | 0 |
| Previous experience with AI-based DSSs | 0 | 2 | 1 | 6 | 4 | 6 | 17 |
| Promoting of competencies in operating with AI-based DSSs | 0 | 3 | 8 | 7 | 8 | 6 | 4 |
| Reduction of uncertainties | 0 | 6 | 6 | 4 | 6 | 10 | 4 |

Table A9: Percent positions and Garret value of user-related factors

| **Rank** | **100(Rij-0,5)/N** | **Percent position** | **Garret value** |
| --- | --- | --- | --- |
| 1 | 100(1-0,5)/36 | 1,39 | 91 |
| 2 | 100(2-0,5)/36 | 4,17 | 83 |
| 3 | 100(3-0,5)/36 | 6,94 | 79 |
| 4 | 100(4-0,5)/36 | 9,72 | 75 |
| 5 | 100(5-0,5)/36 | 12,50 | 72 |
| 6 | 100(6-0,5)/36 | 15,28 | 70 |
| 7 | 100(7-0,5)/36 | 18,06 | 68 |

Table A10: Calculation of Garret score and ranking of user-related factors

| **Factors** | **Rank 1* 91** | **Rank 2*83** | **Rank 3*79** | **Rank 4*75** | **Rank 5*72** | **Rank 6*70** | **Rank 7*68** | **Total** | **Score (Total/N)** | **Ranking** |
| --- | --- | --- | --- | --- | --- | --- | --- | --- | --- | --- |
| Knowledge and understanding of how AI-based systems work | 637 | 249 | 395 | 525 | 360 | 350 | 272 | 2788 | 77,44 | 4 |
| Perceived added value of the use of AI-based DSSs | 1274 | 415 | 474 | 300 | 288 | 70 | 136 | 2957 | 82,14 | 1 |
| Openness of potential users | 637 | 498 | 237 | 450 | 360 | 280 | 340 | 2802 | 77,83 | 3 |
| Trust in the functioning of AI-based DSSs | 728 | 913 | 553 | 150 | 288 | 280 | 0 | 2912 | 80,89 | 2 |
| Previous experience with AI-based DSSs | 0 | 166 | 79 | 450 | 288 | 420 | 1156 | 2559 | 71,08 | 7 |
| Promoting of competencies in operating with AI-based DSSs | 0 | 249 | 632 | 525 | 576 | 420 | 272 | 2674 | 74,28 | 6 |
| Reduction of uncertainties | 0 | 498 | 474 | 300 | 432 | 700 | 272 | 2676 | 74,33 | 5 |
