# Supplementary material for: Implementation of artificial intelligence-based decision support systems for antibiotic prescribing in hospitals: a Delphi study
Source: Front Digit Health. 2025 Apr 25;7:1555042. doi: 10.3389/fdgth.2025.1555042 (PMC12062133; doi:10.3389/fdgth.2025.1555042)
Supplement: Supplementary file 3 [file Table3.docx]

Additional File 3

Table A1: Assessment of the realization of factors (n=36)

| **Factors** | **Very good realized** | **Rather good realized** | **Rather poorly realized** | **Poorly realized** | **Not realized at all** | **I cannot assess** |
| --- | --- | --- | --- | --- | --- | --- |
| **Technological factors** | | | | | | |
| Clear presentation of the results | 2,8% | 11,1% | 13,9% | 11,1% | 19,4% | 41,7% |
| Reliable database | 5,6% | 8,3% | 11,1% | 2,8% | 16,7% | 55,6% |
| System integration/ compatibility | 2,8% | 13,9% | 11,1% | 13,9% | 22,2% | 36,1% |
| Existence of warning functions | 5,6% | 16,7% | 19,4% | 11,1% | 11,1% | 36,1% |
| Existence of alternative suggestions | - | 16,7% | 8,3% | 11,1% | 22,2% | 41,7% |
| Assurance of data security | 11,1% | 16,7% | 8,3% | - | - | 63,9% |
| Precise recommendations | 8,3% | 19,4% | 8,3% | 16,7% | 13,9% | 33,3% |
| Manageable user interface with easy navigation | - | 16,7% | 19,4% | 11,1% | 16,7% | 36,1% |
| Rapid system updating | 5,6% | 11,1% | 11,1% | 11,1% | 8,3% | 52,8% |
| Automated data transfer | 2,8% | 5,6% | 11,1% | 13,9% | 19,4% | 47,2% |
| Easy access to the system/ data | 2,8% | 13,9% | 19,4% | 13,9% | 13,9% | 36,1% |
| Traceability of recommendations | - | 16,7% | 19,4% | 16,7% | 13,9% | 33,3% |
| Completeness of recommendations | 5,6% | 11,1% | 16,7% | 11,1% | 19,4% | 36,1% |
| Easy manual data entry | 2,8% | 13,9% | 11,1% | 11,1% | 16,7% | 44,4% |
| Individual-specific recommendations | 5,6% | 5,6% | 8,3% | 19,4% | 22,2% | 38,9% |
| **Factors** | **Very good realized** | **Rather good realized** | **Rather poorly realized** | **Poorly realized** | **Not realized at all** | **I cannot assess** |
| **Organizational factors** | | | | | | |
| Promotion of the hospital's willingness to change | - | 22,2% | 13,9% | 5,6% | 16,7% | 41,7% |
| Restructuring of “traditional” working routines | - | 5,6% | 8,3% | 13,9% | 16,7% | 55,6% |
| Promotion of the openness of (medical) teams/units | 5,6% | 11,1% | 19,4% | 2,8% | 16,7% | 44,4% |
| Assurance of technical equipment | 5,6% | 16,7% | 13,9% | 25,0% | 11,1% | 27,8% |
| Assurance of technical support | 5,6% | 13,9% | 13,9% | 16,7% | 16,7% | 33,3% |
| Clarification of the legal framework | 2,8% | 8,3% | 8,3% | 11,1% | 13,9% | 55,6% |
| Restructuring medical education | 2,8% | 5,6% | 11,1% | 11,1% | 27,8% | 41,7% |
| Assurance of support from the management level | 2,8% | 11,1% | 5,6% | 16,7% | 13,9% | 50,0% |
| Users´ participation in the development and implementation phase | 2,8% | 11,1% | 19,4% | 8,3% | 16,7% | 41,7% |
| Training of potential users | 5,6% | 13,9% | 8,3% | 16,7% | 13,9% | 41,7% |
| Overcoming hierarchical structures | 8,3% | 2,8% | 11,1% | 16,7% | 22,2% | 38,9% |
| **Factors** | **Very good realized** | **Rather good realized** | **Rather poorly realized** | **Poorly realized** | **Not realized at all** | **I cannot assess** |
| **User-related factors** | | | | | | |
| Promotion of competencies in operating with AI-based DSSs | - | 8,3% | 19,4% | 11,1% | 27,8% | 33,3% |
| Reduction of uncertainties | - | 5,6% | 19,4% | 16,7% | 25,0% | 33,3% |
| Promotion of the openness of potential users | - | 16,7% | 13,9% | 11,1% | 22,2% | 36,1% |
| Sharing of Knowledge and understanding of how AI-based systems work | 2,8% | 2,8% | 16,7% | 19,4% | 25,0% | 33,3% |
| Showing the added value of the use of AI-based DSSs | - | 13,9% | 19,4% | 11,1% | 22,2% | 33,3% |
| Promotion of trust in the functioning of AI-based DSSs | - | 11,1% | 19,4% | 16,7% | 22,2% | 30,6% |
| Facilitating experience with AI-based DSSs before actual use | - | 5,6% | 11,1% | 16,7% | 30,6% | 36,1% |
